# Being Single as a Social Barrier to Access Reproductive Healthcare Services by Iranian Girls

**DOI:** 10.15171/ijhpm.2016.107

**Published:** 2016-08-17

**Authors:** Shahnaz Kohan, Fatemeh Mohammadi, Firoozeh Mostafavi, Ali Gholami

**Affiliations:** ^1^Nursing and Midwifery Care Research Center, Faculty of Nursing and Midwifery, Isfahan University of Medical Sciences, Isfahan, Iran.; ^2^Student Research Center, Faculty of Nursing and Midwifery, Isfahan University of Medical Sciences, Isfahan, Iran.; ^3^Department of Health Education and Promotion, Faculty of Health, Isfahan University of Medical Sciences, Isfahan, Iran.; ^4^Department of Islamic Sciences, Isfahan University of Medical Sciences, Isfahan, Iran.

**Keywords:** Reproductive Healthcare, Women, Culture, Qualitative Study

## Abstract

**Background:** Iranian single women are deprived of reproductive healthcare services, though the provision of such services to the public has increased. This study aimed to explore the experiences of Iranian single women on their access to reproductive health services.

**Methods:** A qualitative design using a conventional content analysis method was used. Semi-structured interviews were held with 17 single women and nine health providers chosen using the purposive sampling method. ****

**Results:** Data analysis resulted in the development of three categories: ‘family’s attitudes and performance about single women’s reproductive healthcare,’ ‘socio-cultural factors influencing reproductive healthcare,’ and ‘cultural factors influencing being a single woman.’ ****

**Conclusion:** Cultural and contextual factors affect being a single woman in every society. Therefore, healthcare providers need to identify such factors during the designing of strategies for improving the facilitation of access to reproductive healthcare services.

## Introduction

The utilization of healthcare services is influenced by healthcare policies, people’s educational level, gender, socio-cultural factors, patterns of disease severity, income level, and access to the services.^1-3^

According to the studies conducted on the utilization of healthcare services, socio-cultural factors are the key determinants of individuals’ willingness to utilize healthcare services.^4^ A vast range of socio-cultural factors influences the utilization of healthcare that corresponds to each country’s culture and context. Therefore, different approaches are required to improve access to reproductive and sexual healthcare by social groups with different demographic characteristics.^5^ Social norms can also marginalize some subgroups such as single women and hinders their access to healthcare services.^6^ Although the number of single women and women in the reproductive age is increasing over the world,^7^ being single in adult age is considered against the social norm in different societies. It is believed that social norms and negative social attributes affect single women’ lives and their general and reproductive health status.^8,9^

### Background in Iran

Many efforts have been made in Iran to provide an equitable distribution of primary healthcare facilities to people. There is also an emphasis on the provision of comprehensive healthcare services to Iranian citizens.^10^ In recent years, supplementary healthcare programs such as the Iranian women’s health, Iranian men’s health and older people’s health have covered various age and gender groups. Reproductive healthcare services as the key element of general healthcare have been added recently to the above-mentioned programs.^11-13^ In addition to the public healthcare services provided by the Iranian government, the private sector also plays an important role in the provision of reproductive healthcare services to the general population.^14^ The improvement of access to and facilities of reproductive healthcare services have increased the utilization of these services by the Iranian society. Nevertheless, single women have been ignored due to the specification of reproductive healthcare services to married women. Furthermore, despite the need to conducting studies on women with different socio-demographic characteristics,^15^ most Iranian studies have focused on married women and adolescent females as their samples and therefore, have missed the reproductive healthcare issues of single women.

Therefore, this study aimed to explore the experiences of Iranian single women on their access to reproductive healthcare services.

Similar to other part of the world, the celibacy of Iranian girls especially in big cities has been increased in recent decades^16^ due to a higher educational level, women’s employment and urbanization.^17^ With an average age of 25 years at the verge of marriage,^16^ Isfahan city has a high rank among other cities in terms of the number of single women who are at the verge of marriage.^18^ About 30% of young adults live alone and it has been said that single women’ inclination to live alone in big cities has increased comparing to the past.^19^ However, living alone has not been yet accepted as a norm in the Iranian society.^20^

Single women live for more years with their families and are expected to behave under the family’s laws. A large percentage of families allows their girls to have sufficient freedom to go to the college, be employed and have free movements in the society. On the other hand, some girls face with various restrictions imposed by their families due to the social norm of getting married at young ages. It is noted that problems associated with financial independence and autonomy are the concerns of many Iranian single women.^21^

## Methods

A qualitative design using a conventional content analysis method was used.

### Research Participants

Participants were consisted of seventeen single women aged between 25 and 60 years old who were the inhabitants of Isfahan city, Iran. Also, nine reproductive healthcare providers working in the public and private sectors were recruited. The participants were chosen using purposive sampling. The inclusion criteria for the selection of the single women were: age between 25 and 60 years and having no history of legal marriage. The exclusion criteria were the participants’ unwillingness to participate in this study. The inclusion criterion for the selection of healthcare providers were having at least 6 months experience of work as reproductive healthcare providers.

To recruit the participants, one of the authors referred to healthcare centers and requested to be provided with the contact information of those families who had a single women as a family member and also met the above-mentioned inclusion criteria. The probable samples were contacted and informed of this study’s purpose and method. Those participants who accepted to take part in this study determined the convenient time and place for holding interviews.

### Data Collection

Face to face semi-structured interviews were held in the participants’ work places, healthcare centers, and educational centers. The focuses of the interviews’ questions were: What do you do, when you encounter any issue related to reproductive health? and What factors facilitate or hinder you to access and utilize reproductive healthcare services? On average, the interviews lasted between 15 and 65 minutes.

### Data Analysis

The method of conventional content analysis suggested by Graneheim and Lundman^22^ was use to analyze data. According to this method, researchers created codes and categories to answer the study’s question. The following steps were taken to analyze data:

- Each interview was recorded and transcribed *verbatim* and read several time to get the sense of whole;
- Meaning units, condensed meaning units, and codes were developed; and
- Through the process of reduction of data, categories and subcategories were developed from the codes.^22^

### Rigor

To ensure of this study’s rigor, credibility, dependability, transferability, and confirmability were considered.^23^ Conducting in-depth interviews at various times and places convenient to the participants, choosing the participants with the consideration of maximum variation and verifying the extracted codes by the participants were in line with ensuring credibility. Also, a couple of healthcare researchers who were familiar with qualitative data analysis approaches were asked to check the analysis process that led to the improvement of this study’s dependability. To assess transferability, a couple of single women was provided with a brief report of data analysis and transcriptions and was asked to confirm that the study’s results were transferable to their own context. The process of data coding and analysis were checked and verified by peer reviewers to ensure of the data analysis’ confirmability.

## Results

In total, 17 single women and nine reproductive healthcare providers consisted of seven midwives and two gynecologists participated in this study. Most single women were between 30 and 40 years old, were employed and had an academic degree education (Table 1). Most of the reproductive healthcare providers were older than 30 years old and had a bachelor degree in midwifery.

**Table 1:** Demographic Characteristics of the Study Participants

**Characteristics**	**Number**
Age	
25-30	6
31-40	7
41-50	2
51-60	2
Educational level	
Primary school	2
Secondary school	2
Diploma	2
Bachelor	9
Master of sciences and higher	2
Occupation	
Employed	9
Unemployed	8

Three categories were developed during the data analysis: ‘family’s attitudes and performance about single women’s reproductive healthcare,’ ‘socio-cultural factors influencing reproductive healthcare,’ and ‘cultural factors influencing being a single woman.’ Each category was consisted of a couple of subcategories described as follows (Table 2).

**Table 2:** Categories and Subcategories Developed During the Data Analysis

**Category**	**Subcategory**
Family’s attitudes and performance about single women' reproductive healthcare	Influence of the family on the formation of girls’ attitudes toward reproductive health-related issues
	Mothers’ ability in the surveillance of their daughters’ reproductive health
Socio-cultural factors influencing reproductive healthcare	Public prejudgment toward reproductive health-related issues
	Silence and sham when faced with reproductive health-related issues
Cultural factors influencing being a single woman	Single women’s autonomy
	Issues related to the preservation of girls’ virginity
	Single women’s literacy about reproductive healthcare
	Stigma toward the utilization of reproductive healthcare services

### Family’s Attitudes and Performance About Single Women’s Reproductive Healthcare 

This category was consisted of two subcategories: ‘Influence of the family on the formation of girls’ attitudes toward reproductive health*-*related issues,’ and “Mothers’ ability in the surveillance of their daughters’ reproductive health.” Individuals’ attitudes influence their behaviors in reproductive healthcare and families were considered important contributors to the formation of girls’ attitudes about healthy behaviors.

### Influence of the Family on the Formation of Girls’ Attitudes About Reproductive Health-Related Issues

The participants stated that public attitudes about reproductive healthcare influenced their decisions to access and utilize reproductive healthcare services. If single women would be taught to consider their reproductive healthcare status as a component of their general health, and would deal with it similar to physiological healthcare, they decided to refer to reproductive healthcare centers without being shameful for dealing with it.

One single participant who was 34 years old stated: “*I feel comfortable when I am referred to the physician for receiving reproductive healthcare services, because I feel that it is a natural biological subject. It is very silly to think that my reproductive health-related issues are embarrassing. For me, it is as if somebody has a stomachache and is ashamed of disclosing his/her pain to his/her family and physician. Of course, I am fully aware of the fact that such issues still have not been comprehensively conceptualized in our society*.”

A gynecologist said: “*In our community, any issue and illness that is related to the reproductive system is considered a sexual problem. So, from a cultural perspective, this disease is deemed obscene and talking and action about it becomes very difficult.*”

The social acceptability of reproductive healthcare was a component of the individuals’ general health status and the only motivation factor that affected the single women’s behaviors. It also had a relationship with reproductive health and sexual issues. Single women should learn to differentiate between the reproductive system diseases and sexual-related issues. If the norm of a society dictated that single women should not engage in sexual relationships and reproductive system’s diseases only resulted from having a sexual relationship, single women refrained from the utilization of reproductive healthcare services.

A 38-year-old single woman said: “*I speak comfortably with my mother about the complications of my reproductive health status, since I think this issue is not unethical or immoral and there is no need to conceal it*.”

### Mothers’ Ability in the Surveillance of Their Daughters’ Reproductive Health

Besides the single women’s need to have an appropriate attitude about reproductive health-related issues, mothers should be able to communicate appropriately with their daughters about such issues from childhood. Therefore, single women would be able to comfortably discuss and disclose complications to them. Such a relationship was required to prevent from the creation of stressful and confusing attitudes about reproductive healthcare in single women and facilitate their referral to reproductive healthcare centers.

A 38-year-old single woman said: “*Once I had a reproductive tract infection, I really wanted to disclose it to my mother, because the infection made me very uncomfortable, but I told about it to my sister instead. Generally, I feel more comfortable to disclose such issues to my elder sister. I have not been comfortable discussing these issues with my mother at all, since I always feel that I am not comfortable enough to discuss these issues with my mother*.”

According to the participants, appropriate mother-daughter communication would not solely facilitate the utilization of reproductive healthcare services by single women, but mothers should play an active role to discover the reproductive health-related complications of their daughters and support them emotionally.

A 40-year-old single woman with regard the role of her mother in her reproductive healthcare status said: “*I referred several times to the gynecologist’s office. Every time that I felt a reproductive complication, I told about it to my mother and she would tell me to see a doctor and accompanied me to the physician’s office. I disclosed my problems to the doctor because the presence of my mother gave me confidence. It was as if my mother was my companion*.”

### Socio-Cultural Factors Influencing Reproductive Healthcare

This category was consisted of two subcategories: ‘public prejudgment toward reproductive health-related issues’ and ‘silence and sham when faced with reproductive health-related issues.’ The participants described the notion of reproductive health-related issues as a taboo and the problem of girls’ silence and shame in disclosing reproductive health-related issues. The utilization of reproductive healthcare services was under the intensive effectuality of the socio-cultural environment, in which the family was a constitutive component. Therefore, the single women faced many challenges when they needed to use reproductive healthcare services.

### Public Prejudgment Toward Reproductive Health-Related Issues

The participants indicated that making reproductive health-related issues as taboo was ingrained upon the girls’ psyche from childhood. This uncomfortable feeling in women was gradually internalized and negatively affected the utilization of reproductive healthcare services.

A 35-year-old single participant expressed: “*When some of my friends of the same age asked questions regarding sexual issues, everybody ignored them with the excuse of their social flippancy and impudence. Every girl who asked about it would be labeled as presumptuous and impudent. Even my own mother called it impolite expressing disgust over such remarks. Therefore, I did not like to ask anything about sexual and reproductive healthcare issues from my parents.*”

The assumptions of reproductive healthcare issues as taboo and socially unpleasant was not only limited to the childhood period, but also were considered obscene for single women as well and prevented them from accessing reproductive healthcare services.

A 58-year-old single participant said: “*My family believes that single individuals should not talk about this stuff at all.”*

It appeared that looking at the reproductive and sexual issues as taboo was not limited to the individual and family level only, but it was spread to educational organizations and media. The unanimous implementation of this assumption made it impossible to educate the public about reproductive health. Consequently, a lack of formal instructive courses about reproductive healthcare resulted in the utilization of unreliable sources such as friends and websites by the single women.

A 32-year-old single participant said: “*The number of single individuals is increasing. They have insufficient information regarding reproductive and sexual issues and some of them do not have any knowledge about them at all. It is incumbent to instruct these issues to this demographic group of the society. Due to religious restrictions, reproductive healthcare is not an issue that can be discussed on state media. For instance, no one can talk about mammography on the television. These issues should be instructed to target groups by other means. Unfortunately, nothing particular has been planned for reaching and educating these target subgroups who have sexual and reproductive healthcare concerns.”*

### Silence and Sham When Faced With Reproductive Health-Related Issues

The social attitudes about reproductive and sexual issues caused the vogue silence and shame in single women in relation to reproductive health-related issues. The participants indicated that they could not discuss their sexual and reproductive complications with their family members.

A 31-year-old single participant said: “*It is difficult to talk about sexual and reproductive healthcare issues to the family. We have been trained not to express our reproductive system concerns and complications openly. Any conveyance of such complications should be done surreptitiously. Therefore, I could not disclose these issues to my family members*.”

Although it seemed that the expression of shame and embarrassment felt in relation to reproductive and sexual issues was exclusive to single women, most participants believed that a sense of reluctance and prudence affected their mothers. They believed that mothers experienced shame and embarrassment during discussion with their daughters about reproductive healthcare. Therefore, their health issues were hidden from their family members especially male members.

A 29-year-old single participant told: “*I think my mother does not feel comfortable discussing these issues with me. For instance, when she wanted to explain menstruation to me, she did it with difficulty. She evaded the topic completely and with agony*.”

Prudence, silence, and secrecy regarding reproductive healthcare issues were observed in the behaviors of healthcare staff.

A 29-year-old single participant said about the behavior of a pharmacy staff: “*When I went to purchase L.D pills, while attempting to give the pills, a worker in the pharmacy was acting surreptitiously, as if he were giving me some illegal drugs. It did not cross his mind that it is his responsibility to explain the efficient way of administering the pills. It is unfair to be treated so, since single women or married women need to use such pills*.”

A midwife remarked on the social concealment of reproductive and sexual issues: “*The levels of social concealment of sexual and reproductive healthcare issues are fairly high. When the cultural look at the problem is corrected, individuals can recognize the right solution more comfortably. However, unhealthy concealments enforce the person with sexual and reproductive complications to choose a wrong solution for solving her health-related issues.”*

### Cultural Factors Influencing Being a Single Woman

Unique cultural circumstances constituted another important factor influencing the utilization of reproductive healthcare services by single women. In this study, being single affected the participants’ autonomy, the preservation of their virginity and reproductive health literacy.

### Single Women’s Autonomy

Iranian single women mostly live with their own family until marriage. It was revealed that single women’s dependency to their family members was a barrier to access reproductive healthcare services. In other words, independent decisions made by the girls and being able to afford healthcare’s expenses facilitated the utilization of reproductive healthcare services by single women.

The movements of the single women in the society were controlled by their family members. Therefore, they felt a low level of autonomy to access reproductive healthcare services.

A 58-year-old single women as an indoor girl who was asked to come to the healthcare center to participate in the study stated: “*When I intended to refer to the healthcare center, I was questioned at home intensively on what my business was in such a center. I became very embarrassed by their questions. I answered that I was going for my mother’s medical reports. Even my brother’s wife followed me to find out the reason behind my referral to the healthcare center.*”

Those single women who had a job or could move in the community lonely, had a better access to reproductive healthcare services.

One specialist stated: “*An indoor girl was brought to the center by her mother and it was her mothers who explained the girl’s problems. On the other hand, educated and outdoor girls who are socially active and employed come to the healthcare centers on their own and explain their problems themselves.”*

A 35-year-old single woman and a teacher shared her story about her visit to a gynecologist: “*When I experienced some problem with my menstrual period, I decided to go to a doctor’s office by myself, when I said my decision at home, no one asked me about it. So I referred to a gynecologist’s office easily.”*

### Issues Related to the Preservation of Girls’ Virginity

The participants expressed that single women’ virginity was important in the girls’ singlehood period and could become a barrier to the efficient utilization of reproductive healthcare services by them. The fear of damaging their virginity during physical examination in healthcare centers created a strong sense of anxiety when they accessed healthcare services.

A 35-year-old single participant said: “*I do my best not to refer to any healthcare center, since I always think that during physical examination, my virginity may be damaged*.”

It is worth mentioning that because of being afraid of the legal consequences of damaging girls’ virginity, even healthcare staff faced challenges for providing reproductive healthcare services to such referents. They tried to refer the single women to other centers.

A midwife stated: “*Sometimes single women do not inform us of their marital status, and they want to put the blame of damaging their virginity on healthcare staff. Such girls use legal authorities to press charges. We are scared in distasteful circumstances and do not feel comfortable to provide healthcare services to single women*.”

Healthcare staff had insufficient knowledge about issues affecting the virginity of single women. Under such circumstances, healthcare staff were incapable of administrating usual and appropriate examinations and therefore, utilized higher technologies with lower availability levels and higher costs, which could be regarded as barriers to the utilization of reproductive healthcare services.

One gynecologist commented: “*Problems arise when I want to do internal vaginal examination. If I doubt about the existence of tumor or any medical complication in the cervix, I face serious problems. I must refer single women to other healthcare centers where they can perform vaginal examinations by hysteroscopy through delicate guides to prevent damaging their hymen. In some cases, they are referred to other centers to perform transrectal sonography; the number of these centers are limited in this city. These technical limitations make some difficulties in the utilization of reproductive healthcare services provided to single women.*”

### Single Women’s Literacy About Reproductive Healthcare

Provision of information and education about reproductive healthcare services to single women were restricted. Therefore, in comparison with married women, single women had low reproductive health literacy. The single women attributed their low literacy levels of reproductive healthcare services to a lack of formal education.

One midwife stated: “*For example, a 35-year-old single woman with breast pain or fibrocystic complications is ashamed of being examined clinically. The reason of this feeling is having no knowledge about the necessity of doing routine breast examinations*.”

It is worth mentioning that single women were not informed about the existence of reproductive healthcare services in healthcare centers. Therefore, individuals became incapable of recognizing and managing their complications and did not know how to use appropriate services, when they faced any complication.

A 35-year-old single woman stated about a lack of proper education in this way: “*Except for vaccination, I never referred to any healthcare center. After my vaccination, I thought that I would have nothing to do with such centers and only children and pregnant women should refer to such places. I never referred there, since my knowledge of such services was low.”*

A 30-year-old single woman stated: “*I do not know where to go when a complication occurs to me. I think the main reason for such low literacy of reproductive healthcare services is a lack of provision of appropriate education. I am sure that there are lots of single women like me who are unaware of the existence of such services in healthcare centers*.”

### Stigma Toward the Utilization of Reproductive Healthcare Services

Legal, religious, and ethical prohibitions regarding pre-marital sexual relationships made the single women to feel the anxiety of stigmatization in the society. Almost all participants believed that one of the main reasons for not patronizing healthcare centers was the abnormality of single women’s referral to reproductive healthcare services from the social perspective.

Some single women avoided the utilization of reproductive healthcare services in both private and public centers.

A 35-year-old single participant stated: “*In our society, there are preconceptions that gynecologists should only provide services to married women, and that single women have nothing to do with them. Therefore, single women feel embarrassed, if they refers to gynecologists. It means that girls do not refer to such centers to avoid stigmatization*.”

Surreptitious and confidential references constituted the single women’s defense mechanism against stigmatizations. These girls did not patronize healthcare centers where the confidentiality of their personal information was not guaranteed.

One midwife stated: “*In healthcare centers, after recording the referent’s name and type of required healthcare service, the services are provided. For this reason, single women are concerned about having their personal information disclosed in such centers. Therefore, they do not patronize healthcare centers and prefer to visit healthcare staff in private clinics*.”

## Discussion

The results of this study showed that barriers and facilitators affecting the single women’s access to reproductive healthcare services were consisted of family’s attitudes and performance about single women’s reproductive healthcare issues, socio-cultural factors influencing reproductive healthcare and cultural factors influencing being a single woman.

Reproductive health-related issues are considered taboos in some cultures.^24,25^ This study reported shame and silence when the single women and healthcare staffs faced reproductive healthcare issues at personal, family, and social levels.^26^ Families were shameful of providing their girls with essential information regarding sexual and reproduction issues. Nevertheless, those families that overcomed the existing cultural barriers, a true and efficient attitude about single women’s reproductive health status emerged and mothers played their supportive roles.

The results of this study indicated that being single brought a limitation to the single women’ autonomy and was a barrier to access reproductive healthcare services. The relationship between girls’ autonomy and the utilization of healthcare services is reported by antecedent studies.^27,28^ A low level of reproductive health literacy and unawareness of available services were other barriers to access reproductive healthcare services stated by the participants.^29,30^

The common fear in the single women and healthcare providers were the damage to the girls’ virginity during clinical examinations in healthcare settings. Other studies reported that avoiding sexual relationships and the maintenance of virginity were considered moral values for girls in the Iranian society.^25,31^ In addition, according to the Iranian law, those healthcare providers that damage single women’ virginity are published legally and financially, even if it is accidental and happens during medical checkups.^32^ Because of technical limitations in administrating routine examinations, the healthcare staffs referred single women to other healthcare centers.

Socio-cultural sensitivities around sexual and reproductive healthcare are higher for vulnerable groups of the society including single women.^14,33^ For instance, if a girl is diagnosed with a reproductive system disease, she is accused of having an illegal sexual relationship by the society. Such a prejudice leads to the stigmatization of single women.^34^ Various studies indicate that stigmatization anxiety against diseases such as AIDS, reproductive system infections and psychological disorders are barriers to receiving healthcare services.^1,35,36^ Therefore, ensuring of the confidentiality of single women’ referral to reproductive healthcare services plays a key role in efficient utilization of healthcare services.^37,38^

## Limitations

The generalization of the findings of this study should be done with caution due to qualitative approach and sample size. This limitation was tried to be reduced through the selection of the participants with maximum variations in demographic characteristics in terms of age, educational level, employment and socio-economic status.

## Conclusion

Socio-cultural context influences Iranian single women’ behaviors in relation to the access and utilization of reproductive healthcare services. Therefore, healthcare providers need to identify such factors during the designation of strategies for improving the facilitation of access to reproductive healthcare services.

## Ethical issues

The research protocol was approved by the research committee affiliated with Isfahan University of Medical Sciences, Isfahan, Iran (No. 392478). The researcher introduced herself to the participants, explained the study’s aim, anonymous identity of their participants and possibility of withdrawal from the study at any time without being penalized. Also, the permission to tape-record the interviews was obtained from them. Finally, of those who agreed to participate in this study written informed consents were obtained.

## Competing interests

The authors declare that they have no competing interests.

## Authors’ contributions

Study conception and design, analysis and interpretation of data, and critical revision: SK, FaM, FiM, and AG; Acquisition of data and Drafting of manuscript: SK and FaM.

## Authors’ affiliations

^1^Nursing and Midwifery Care Research Center, Faculty of Nursing and Midwifery, Isfahan University of Medical Sciences, Isfahan, Iran. ^2^Student Research Center, Faculty of Nursing and Midwifery, Isfahan University of Medical Sciences, Isfahan, Iran. ^3^Department of Health Education and Promotion, Faculty of Health, Isfahan University of Medical Sciences, Isfahan, Iran. ^4^Department of Islamic Sciences, Isfahan University of Medical Sciences, Isfahan, Iran.

## Key messages

**Implications for policy makers**

- Despite the development and widespread utilization of reproductive healthcare services in Iran, single women have a limited access to these services.
- To increase the utilization of reproductive healthcare services by single women, contextual factors should be considered and incorporated into healthcare programs.
- Appropriate interventions are required for the modification of individual, interpersonal, and socio-cultural issues that hinder single women’ access to reproductive healthcare services.
- Limited health literary, the family’s poor attitudes and functions, socio-cultural issues and being a member of a specific social group are barriers to access reproductive healthcare services by Iranian single women.

**Implications for public**

Single women as a marginalized group in Iran do not have sufficient access to reproductive healthcare services due to cultural taboos and public prejudices. In this respect, culture making for the acceptation of reproductive healthcare services as an integral part of general healthcare is required. Also, families need to be educated about childrearing and importance of single women’ reproductive healthcare. In addition, the fear of families of losing girl’s virginity during visits by healthcare professionals should be addressed. Families should be informed that vaginal examination is not in the provision of reproductive healthcare services. Lastly, single women need to be empowered to be self-care agents in reproductive health.
